# Mathematical Structures in Group Decision-Making on Resource Allocation Distributions

**DOI:** 10.1038/s41598-018-37847-2

**Published:** 2019-02-04

**Authors:** Noah E. Friedkin, Anton V. Proskurnikov, Wenjun Mei, Francesco Bullo

**Affiliations:** 1 University of California Santa Barbara, Department of Sociology and Center for Control, Dynamical-Systems and Computation, Santa Barbara, California 93106 USA; 2 Delft University of Technology, Delft Center for Systems and Control, Delft, 2628 CD Netherlands; 3 Institute for Problems in Mechanical Engineering of the Russian Academy of Sciences, St. Petersburg, 199178 Russia; 4 ETH Zürich, Automatic Control Laboratory, 8092 Zurich, Switzerland; 5 University of California Santa Barbara, Department of Mechanical Engineering and Center for Control, Dynamical-Systems and Computation, Santa Barbara, California 93106 USA

## Abstract

Optimal decisions on the distribution of finite resources are explicitly structured by mathematical models that specify relevant variables, constraints, and objectives. Here we report analysis and evidence that implicit mathematical structures are also involved in group decision-making on resource allocation distributions under conditions of uncertainty that disallow formal optimization. A group’s array of initial distribution preferences automatically sets up a geometric decision space of alternative resource distributions. Weighted averaging mechanisms of interpersonal influence reduce the heterogeneity of the group’s initial preferences on a suitable distribution. A model of opinion formation based on weighted averaging predicts a distribution that is a feasible point in the group’s implicit initial decision space.

## Introduction

Resource allocation decisions occur in every institutional sector of society; for example, in government, education, health care, commerce, philanthropic, and military organizations among others. The decisions may be routine slow-paced incremental updates of a resource distribution^1,2^, or nonroutine fast-paced responses to unexpected opportunities or problems. They may involve allocations of money, persons, or materials. Although allocation decisions are sometimes directly determined by the votes of a society’s citizens^3^, the more usual basis are small collaborative groups that have been assembled within organizations to deal with particular types of tasks. In such groups, there are two classic modes of decision-making. If the decision-making group agrees on a set of quantifiable task variables, constraints, and objectives, then an optimum resource distribution may be obtained algorithmically^4–8^. The alternative mode is to seek a satisfactory consensus distribution from group discussion. Herbert Simon’s^9,10^ Nobel Prize work on optimum versus satisfactory decisions pointed to two fundamental features of these modes. (i) Both typically involve a *bounded rationality* in which the decision space of all possible distributions is not considered, but instead are constrained to a subspace of possibilities. (ii) Neither one is objectively superior to the other. Decisions are either obtained by “finding optimum solutions for a simplified world” (for example, a world in which distributive justice values and social friction costs are ignored) or by “finding satisfactory solutions for a more realistic world”^10^. If the choice is to formulate an optimization model, then concerns related to realism prompt an effort to quantify a multi-objective definition of a problem that might be solved. If the choice is to base a decision on group discussion, then the effort is oriented toward reaching a consensus on an allocation distribution. Consensus is implicated in both modes. If a group is involved in defining an optimization model, then reaching consensus on its definition (variables, constraints, and objectives) is the precursor of its solution. Flawed decisions may be generated by an unrealistic optimization model or by an informal social process of group deliberation^11–14^. Successful decisions may be generated by both. There is mounting evidence that groups may collectively encode, store, and retrieve knowledge and, thus, usefully exploit the distributed memories and acquired skills relevant to group decision-making^15–19^. We advance this line of research on group decision-making with evidence that points to a natural form of bounded rationality that is related to formal optimization procedures.

If reaching consensus is a desirable feature of resource allocation decisions, then the network science on opinion dynamics may advance our understanding of how groups reach consensus or fail to do so, and why they have settled on a particular problem definition or a resource distribution. The development of networked models of opinion dynamics is now an active interdisciplinary field of research^20–23^ attracting scientists from sociology, mathematics, engineering, physics, and computer science. In general, these models describe a dynamical interpersonal influence system on *n* individuals that is based on three constructs: (i) An array of *n* initial opinions, which can be scalar or multi-dimensional^24–29^. (ii) An influence network of *n* individuals that is a directed weighted graph, where the value *w*_*ij*_ > 0 of arc *i* → *j* stands for the influence weight allocated by individual *i* to individual *j*. (iii) An interpersonal influence mechanism unfolding on the network that specifies how individuals are updating their opinions. The seminal model of the field is the French-Harary-DeGroot system^30–32^

1 $${x}_{i}(k+1)={\sum }_{j=1}^{n}{w}_{ij}{x}_{j}(k),\,\forall \,i=1,\ldots ,n,\,\forall \,k=0,1,2,\ldots $$

With weights *w*_*ij*_ ∈ [0, 1] and ${\sum }_{j=1}^{n}\,{w}_{ij}=1$ ∀*i*, the model stipulates a mechanism of iterative weighted averaging of the opinions^32^. Each positive weight *w*_*ij*_ > 0 corresponds to the direct influence of individual *j*’s opinion on individual *i*, and to the directed $i\mathop{\longrightarrow }\limits^{{w}_{ij}}j$ arc in an influence network of *n* individuals. Individuals in this network may have loops $i\mathop{\longrightarrow }\limits^{{w}_{ii}\mathrm{ > 0}}i$, corresponding to self-weights. When *w*_*ij*_ = 1, the opinion of *j* determines the opinion of *i* at the next period. When *w*_*ij*_ = 0, the opinion of *j* ≠ *i* has no direct influence on the opinion of *i*, but *j* may have indirect influence (for example, $i\,\mathop{\longrightarrow }\limits^{{w}_{ik}}\,k\,\mathop{\longrightarrow }\limits^{{w}_{kj}}\,j$) through other individuals’ opinions. The model is consistent with the formation of consensus on any of the initial opinions or a compromise that is not any of the initial positions. The system is consistent with failures to reach consensus, however, it predicts consensus in any network that has at least one globally reachable node (an individual influencing, directly or indirectly, all other individuals) and satisfies some aperiodicity conditions, e.g. *w*_*ii*_ > 0∀*i*. The Friedkin-Johnsen^26,27,33^ generalization of this model allows for individuals with ongoing attachments to their initial opinions,

2 $${x}_{i}(k+1)={a}_{ii}{\sum }_{j=1}^{n}{w}_{ij}{x}_{j}(k)+(1-{a}_{ii}){x}_{i}(0)\,\forall \,i=1,\ldots ,n,\,k=0,1,2,\ldots ,$$

where *a*_*ii*_ = 1 − *w*_*ii*_ ∀*i*. Besides the Friedkin-Johnsen model, other weighted averaging mechanisms have been proposed, allowing opinion-dependent weights *w*_*ij*_ and other nonlinear structures^21,34,35^. We work with the Friedkin-Johnsen model for several reasons. First, its generalization has been extensively analyzed with proofs of properties and its robustness to relaxations of assumptions^36–38^. Second, its predictions have been empirically evaluated on small groups with complete and incomplete (constrained) communication networks on one-dimensional judgment and truth statement issues^18,27,33,39–41^. Third, the model’s parameters *w*_*ij*_ can be measured in experiments without special procedures of identification (parameter fitting). In this paper, we evaluate the applicability of the Friedkin-Johnsen model from Eq. 2 to opinion dynamics on *multidimensional* resource allocation opinion arrays.

The resource allocation opinion we investigate is a multi-dimensional position **x**_*i*_ = (*x*_*i*1_, *x*_*i*2_, …, *x*_*im*_), *i* = 1, 2, … *n*, that is, a vector of values describing *i*’s preference on the distribution of a supply of *s* units of a resource among *m* options

3 $${x}_{i1}+{x}_{i2}+\ldots +{x}_{im}=s > 0,\,{x}_{ij}\ge 0\,\forall \,i=1,2,\ldots n,\,j=1,\ldots ,m.$$

**Figure 1:**
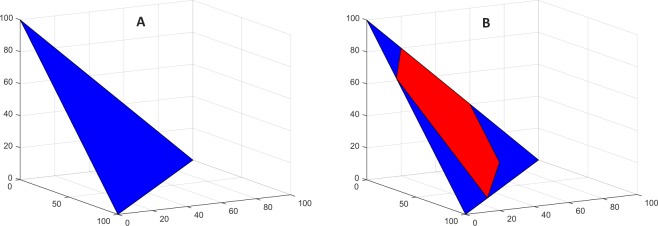
Group generated informal polytope of possible 3-dimensional resource distributions. (**A**) Eq. 3 with *m* = 3 and *s* = 100 defines a triangular plane, and (**B**) the group’s extremal min-max initial distribution preferences on each *j* = 1, 2, 3 cut a polyhedral decision space. The triangular plane (blue) and the set of cuts (red) on it are automatic implicit features of the group’s co-orientation to a resource distribution and its members’ extremal initial min-max preferences on each dimension.

The *n* individuals’ initial distribution preferences **x**_*i*_(0), *i* = 1, 2, … *n*, may differ, and the group may or not reach a consensus on a distribution. This generic Eq. 3 class of resource allocation issues locates opinions on a hyperplane in *m*-dimensional Euclidean space. Figure 1 illustrates: (A) If *m* = 3, then Eq. 3 defines a triangular plane, and (B) the group’s extremal (min-max) initial opinion values on each of the 3 dimensions of the allocation cut a polygonal decision space on the plane. In higher dimensional decision making, the min-max constraints imposed by the initial positions and the Eq. 3 constraint, describe a bounded convex polytope. In other words, whenever a group is deliberating on how to distribute a supply *s* of a resource among *m* options, a polytopic decision space is automatically implicated and defined (implicitly) by the group’s extremal min-max preferences. In optimization models, such polytopes are explicitly constructed by a single individual or group to define a multidimensional space of feasible distributions. In optimization, a distribution is sought that maximizes (minimizes) the value of an explicit objective function. In informal group decision making, opinion dynamics generate a satisfactory settled upon distribution. If the opinion dynamics is based on weighted averaging influence, then the settled upon distribution will be one of the positions in the group’s implicit polytope decision space. Whether such outcomes are a reliable feature of groups’ resource allocation decisions is the subject of this article. Findings from experiments on groups of human subjects are reported on a set of resource allocation issues to which Eq. 3 applies. The experiments begin with a simple optimization problem and progressively relax the formal constraints typically involved in an optimization model. Our findings evaluate the extent to which individuals’ post-discussion settled distribution preferences are constrained by their group’s informal (implicit) automatic polytopes, and the extent to which the Eq. 2 weighted averaging mechanism predicts their settled opinions.

## Results

Findings are reported from five experiments on resource allocation issues. Experiment 1 is a problem on optimum allocation of funds to three categories of work on a device with the objective of maximizing the device performance. This problem is a particular case of linear programming^4–8^ with given quantifiable objectives and given constraints, that is,

4 $$\begin{array}{c}{\rm{m}}{\rm{a}}{\rm{x}}{\rm{i}}{\rm{m}}{\rm{i}}{\rm{z}}{\rm{e}}\,{c}_{1}{x}_{1}+{c}_{2}{x}_{2},+\ldots ,+{c}_{m}{x}_{m}\\ {\rm{s}}{\rm{u}}{\rm{b}}{\rm{j}}{\rm{e}}{\rm{c}}{\rm{t}}\,{\rm{t}}{\rm{o}}\,(a)\,{l}_{1}\le {x}_{1}\le {u}_{1},{l}_{2}\le {x}_{2}\le {u}_{2},\ldots ,{l}_{m}\le {x}_{m}\le {u}_{m};\,(b)\,{x}_{1}+{x}_{2}+\ldots ,{x}_{m}=s,\end{array}$$

where *s* > 0 and (*c*_*j*_, *l*_*j*_, *u*_*j*_), *j* = 1, …, *m*, are known values.

Experiment 2 relaxes the condition of a given objective function. With given constraints (a-b), the task is to attempt to reach consensus on a distribution that satisfies the constraints. Experiment 3 relaxes the condition of a given quantifiable objective and a given (a) set of constraints. The task is to reach a consensus on a set of constraints (a) and then to reach a consensus on a distribution that satisfies all the constraints (a-b). Experiments 4–5 relax the condition of a given quantifiable objective and a set of given or demanded explicit constraints (a). The task is to reach consensus on a distribution that satisfies (b). From the data on experiments 2–5, we evaluate the extent to which individuals’ post-discussion settled distribution preferences are constrained by their group’s informal (implicit) automatic polytopes. Note that in experiment 2, where explicit constraints are given, the group’s implicit polytope (defined by the initial positions) may be a subspace of the explicitly given decision space (defined by the inequalities (a)). We also evaluate the extent to which the Eq. 2 weighting averaging mechanism predicts subjects’ settled distribution preferences and opinion changes. A group’s predicted final opinion(s) are obtained from the matrix equation corresponding to the system of the Eq. 2 scalar equations

5 $${\bf{X}}(k+\mathrm{1)}={\bf{AWX}}(k)+({\bf{I}}-{\bf{A}}){\bf{X}}\mathrm{(0),}\,\,k=\mathrm{0,}\,\mathrm{1,}\ldots ,$$

where the **X**(⋅) matrices are the *n* × *m* arrays of the group’s evolving preferences on a satisfactory distribution, **W** = (*w*_*ij*_) is the matrix of influence weights, and **A** = d*iag* (*a*_11_, …, *a*_*nn*_) is an *n* × *n* diagonal matrix with entries *a*_*ii*_ = 1 − *w*_*ii*_ ∀*i* (the off-diagonal entries are zeros). The predicted final opinions $\hat{{\bf{X}}}$ = **X**(∞) are given by equilibrium equation of the dynamical system

6 $$\begin{array}{c}\hat{{\bf{X}}}={\bf{AW}}\hat{{\bf{X}}}+({\bf{I}}-{\bf{A}}){\bf{X}}\mathrm{(0)}\iff \hat{{\bf{X}}}={\bf{VX}}\mathrm{(0)}\\ {\bf{V}}={({\bf{I}}-{\bf{AW}})}^{-1}({\bf{I}}-{\bf{A}}\mathrm{).}\end{array}$$

where **V** = (*v*_*ij*_) is a row-stochastic matrix (that is, 0 ≤ *v*_*ij*_ ≤ 1 ∀*ij*, ${\sum }_{j\mathrm{=1}}^{n}\,{v}_{ij}=1$ ∀*i*) whose *v*_*ij*_ entries give the total (direct and indirect) influence of *j*’s initial opinions on *i*’s equilibrium opinions. The predicted net opinion changes are given by

7 $$\hat{{\bf{X}}}-{\bf{X}}\mathrm{(0)}={\bf{A}}[{\bf{W}}\hat{{\bf{X}}}-{\bf{X}}\mathrm{(0)]}$$

Eq. 5 is in the class of linear discrete-time dynamical system models with time-invariant {**A**, **W**, **X**(0)} constructs. Recall that **A** is determined by the main diagonal *w*_*ii*_ values of **W**. Laboratory experiments on human subjects allow a measure of its multidimensional **X**(0) array of individual initial opinions, and a measure of the group’s influence matrix **W**. No parameter estimation (optimizing the fit of predictions) is involved. The dynamical system determines the **V** that transforms the group’s multidimensional **X**(0) array of individual initial opinions to a multidimensional $\hat{{\bf{X}}}$ = **X**(∞) array of settled opinions. To evaluate the correspondence of observed and predicted final opinions and opinion changes, the criterion is the strength of the linear correspondence of the stacked observed and predicted final opinions or opinion changes. For **X**(0) with multiple columns **x**^1^, …, **x**^*m*^, the same predictions are generated either with **X**(*k* + 1) = **AWX**(*k*) + (**I** − **A**)**X**(0) or **x**^*j*^(*k* + 1) = **AWx**^*j*^(*k*) + (**I** − **A**)***x***^*j*^(0), *j* = 1, …, *m*. For this reason, we can work with the “column vectorizations” of opinion matrices **X**, stacking the columns of observed and predicted settled opinions or opinion changes on top of one another. Prediction errors must arise either from construct measure errors or misspecification of the influence mechanism. Note that the model’s influence network is defined by the assumed mechanism. The influence network of the group on a specific issue is a cognitive structure assembled by the weights involved in each individual’s weighted-averaging information-integration and update activity.

See Materials and Methods for a description of the subject pools, experimental design, and measurement models. See Supplementary Materials on properties of the Eq. 5 weighted averaging model, and its generalization to multiple or multidimensional issues.

### Experiment 1

An optimization problem was posed to 104 subjects nested 30 groups with 3–4 members:

You are a contractor who has received $10 M dollars to build a device. Your group has been assembled to make a decision on how to allocate this money to three categories of work on the device (Category X, Category Y, and Category Z). You must decide on what fraction of the $10 M to allocate to each category, say x = 0.10 to Category X, y = 0.10 to Category Y, and z = 0.80 to Category Z. These fractions (x + y + z) must sum to 1, so that all of the $10 M is spent. But it matters what fractions you decide upon. Given $10 M that we have to work with, we know that the level of the device’s performance is determined as follows: 1.25x + 1.50 y + 1.75z. There are, however, constraints on the values of x, y, and z: x must be at least 0.45 and at most 0.65, y must be at least 0.10 and at most 0.35, and z must be at least 0.20 and at most 0.35. Given these constraints, we need you to determine what fractions x, y, and, z will give us the highest device performance level.

The optimization problem is to maximize a device-performance function 1.25*x*_1_ + 1.50*x*_2_ + 1.75*x*_3_ under the condition of given constraints (0.45 ≤ *x*_1_ ≤ 0.65, 0.10 ≤ *x*_2_ ≤ 0.35, 0.20 ≤ *x*_3_ ≤ 0.35) and *x*_1_ + *x*_2_ + *x*_3_ = 1. This problem is a particular instance of a linear programming model of Eq. 4. A substantial fraction (34.6%) of the 104 individuals’ initial solutions were incorrect. Consensus was reached in 27 groups, all of which settled on the correct solution. Group discussion markedly reduced the hazard rate of incorrect solutions. More complex optimization problems usually are impossible to solve by hand.

### Experiments 2–3

Experiments 2–3 are motivated by Stigler’s^42^ seminal article on optimal decisions in which he used a diet problem as an illustration and remarked on the difficulties involved in securing the givens required to solve the problem. In his effort to specify its required known values, he was confronted with what he described as “the almost infinite complexity of a refined and accurate assessment of nutritive value of a diet.” We posed two diets problems. (1) Subjects privately recorded their initial positions on the problem. (2) A group discussion was then opened with the instruction: “Discuss the problem with the other members of your group. The goal is to reach an agreement. However, the conversation that you will have may or may not lead you to alter your initial answer, and you may not come to an agreement as a group.”

#### Experiment 2

This experiment relaxes the assumption of a quantifiable objective:

Your group is planning to sail from California to Hawaii. Storage space is limited, so careful planning is required. All food brought aboard the boat must be essential to maintain the health and morale of the group, and acceptable to everyone. We need foods that provide Carbohydrates, Protein, and Fat all which contribute to our daily caloric intake, which must be at least 2,000 calories. Our body needs carbohydrates, protein and fats to fuel its physical activity and metabolic needs. The Food and Nutrition Board of the Institute of Medicine (IOM) provides a range of your total caloric intake that is acceptable for each nutrient. The IOM Acceptable Macronutrient Distribution Range (AMDR) is: 45–65% Carbohydrates, 10–35% Protein, and 20–35% Fat. Any blend (which must sum to 100%) that falls within the IOM’s AMDR will ensure adequate nutrition. What is your preferred blend?

**Figure 2:**
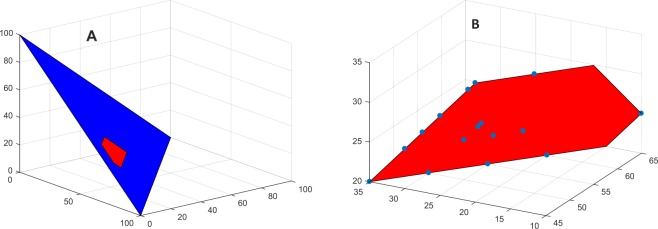
Relaxing the assumption of an explicit objective. An ideal distribution is sought under the condition of given min-max constraints on the permissible values of a 3-dimensional (*x*_1_, *x*_2_, *x*_3_) resource allocation issue. (**A**) With the given min-max constraints (45 ≤ *x*_1_ ≤ 65, 10 ≤ *x*_2_ ≤ 35, 20 ≤ *x*_3_ ≤ 35) a low volume (red) polyhedron decision space is set up that is common to all groups. This common decision space is subject to reduction in different ways by different groups’ min-max initial positions on each dimension of the issue. (**B**) The displayed (blue) points are the final positions of the 80 group members.

This consensus formation problem was posed to 80 individuals nested in 23 groups with 3–4 members. An ideal distribution was sought under the condition of given min-max constraints on the permissible values of a 3-dimensional *x*_1_ + *x*_2_ + *x*_3_ = 100 resource allocation issue. The given min-max constraints (45 ≤ *x*_1_ ≤ 65, 10 ≤ *x*_2_ ≤ 35, 20 ≤ *x*_3_ ≤ 35) set up a low volume polyhedron decision space. Figure 2(A) shows that these given constraints on individuals’ initial positions define a decision subspace (red) of the triangular (blue) space. All groups shared this same (red) low volume decision space. This common decision space is subject to reduction in different ways by different groups’ min-max initial ideal positions on each dimension of the issue. (B) The displayed (blue) points are the final positions of the 80 group members. A consensus on an ideal distribution was reached in 17 of the 23 groups, and 72 (90%) of the 80 individuals’ final positions are located on their group’s informal decision spaces. The linear correspondence (correlation coefficient *ρ*) of observed and weighting-averaging predicted final positions is *ρ* = 0.978, p < 0.0001(t-test), *n* = 240. The linear correspondence observed and weighting-averaging predicted opinion changes is *ρ* = 0.829, p < 0.0001, *n* = 240.

#### Experiment 3

This experiment relaxes both the assumption of a quantifiable objective and given constraints. The problem specification involves a two-part social process in which individuals (i) enter into a group discussion with heterogeneous initial positions on their preferred min-max constraints and their preferred ideal positions, (ii) attempt to reach consensus on a set of constraints, and (iii) attempt to reach consensus on an ideal position that satisfies their agreed upon constraints.

There are 4 food groups from which we can get our essential nutrients: Fruits (e.g., Apples, Apricots, Blueberries, Peaches, Pineapple, Nuts), Vegetables (e.g., Broccoli, Carrots, Corn, Potatoes, Spinach, Peas, Beans), Grains (e.g., Rice, Pasta, Biscuits, Oatmeal, Cereal, Tortillas, Grits), and Meats (e.g., Beef, Turkey, Chicken, Fish). There are no strict guidelines on the minimum and maximum percentages of a daily diet of these food groups. What is your recommendation on the minimum and maximum percent of our total food consumption that should be based on (1) Fruits or Vegetables, (2) Grains, and (3) Meats? What are your “ideal percentages” (which must sum to 100%) in your preferred “At Least to At Most” ranges?”

**Figure 3:**
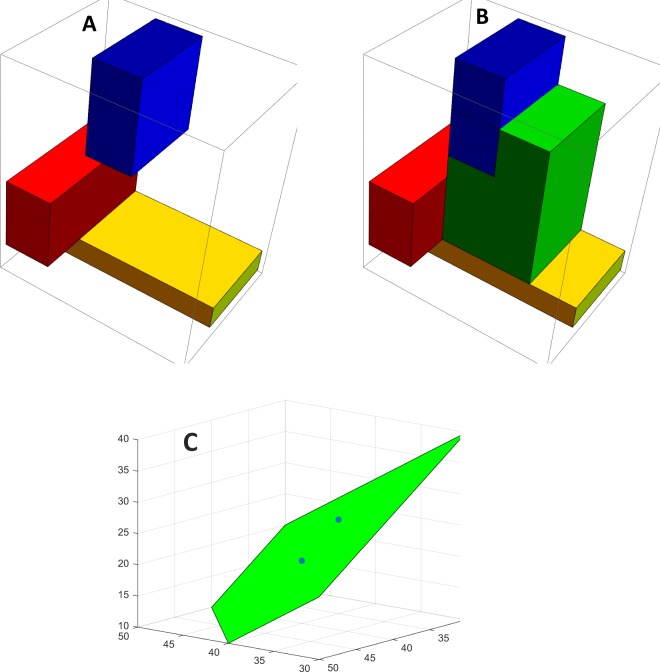
The geometric objects related to a two-part task of reaching consensus on constraints and an ideal position. (**A**) Three individuals in this group have different initial positions on constraints, which correspond to alternative idiosyncratic box decision spaces (blue, red, yellow), and they have different ideal initial positions in their idiosyncratic decision spaces. (**B**) A consensus was reached on min-max constraints, and this agreement sets up the green constraints box. (**C**) The group-specific decision space within the group’s agreed upon constraints box that is a polyhedron cut by the group’s min-max initial ideal positions on each dimension of the resource distribution. The group failed to reach consensus on an ideal position. Two individuals agreed on a final position, different from the third individual’s final position.

This consensus formation problem was posed to 80 individuals nested in 23 groups with 3–4 members (the same individuals who were involved in Experiment 2). The geometric features of the two-part process are illustrated in Fig. 3 on one group of 3 individuals in our data. Figure 3(A) shows that the 3 individuals have different initial positions on constraints, which correspond to alternative idiosyncratic box decision spaces (blue, red, yellow). A consensus was reached on min-max constraints, and this agreement set up the green Fig. 3(B) constraints box. The Fig. 3(C) polyhedron is the implicit group-specific decision space within the group’s agreed upon constraints box that is cut by the group’s min-max initial ideal positions on each dimension of the resource distribution. The group failed to reach consensus on an ideal distribution. Two of the individuals agreed on an ideal position and the third individual dissented. Both final positions are in the group’s implicit polyhedral decision space. For each of the 23 groups, there is an associated group-specific geometry in which groups succeed or fail to reach consensus on constraints or ideal positions. In Part 1 of the experiment on constraints, 68 (85.0%) of the 80 individuals’ final positions on 6 constraint values are on the implicit decision spaces generated by the group’s min-max initial values on constraints. In Part 2 of the experiment on ideal positions, 74 (92.5%) of the 80 individuals’ final positions are on the decision spaces generated by their group’s min-max initial values on an ideal positions. In these data, reaching a consensus on constraints is independent of reaching consensus on an ideal position (Fisher’s exact test *p* > 0.05). In Part 1 of the experiment on constraints, the correspondence observed and predicted final positions is *ρ* = 0.884, p < 0.0001, *n* = 480, and the correspondence of observed and predicted position changes is *ρ* = 0.774, p < 0.0001, *n* = 480. In Part 2 of the experiment on ideal positions, the correspondence of observed and predicted final positions is *ρ* = 0.879, p < 0.0001, *n* = 240, and the correspondence of observed and predicted opinion changes is *ρ* = 0.847, p < 0.0001, *n* = 240.

### Experiments 4–5

These experiments completely relax the condition of any given or demanded explicit constraints. Two consensus formation problems were posed to 118 individuals nested in 34 groups with 3–4 members. Experiment 4 deals with a 3-dimensional distribution issue, and Experiment 5 deals with a 4-dimensional distribution issue

Experiment 4. A large number of positions (permissions to recruit new faculty) are available to allocate to the departments of economics, political science, and sociology. A committee has been formed to provide a recommendation on the allocations, and you are a member of this committee. These positions could be distributed in any way among 3 departments. What percentage of these positions should be allocated to each department? These 3 numbers must sum to 100%. All departments must be allocated some positions. An even distribution of the positions (1/3, 1/3, 1/3) × 100% is possible but unlikely to be adopted.

Experiment 5. If you were a State Legislator what would be your opinion on the percentage of state tax revenues that should be allocated to each the following categories: (i) Spending on Education, (ii) Spending on State Employee Wages, Health Care, and Pensions, (iii) Spending on State Physical Infrastructure Improvements, and (iv) All Other Categories (Welfare, Other Costs of Government, Etc.)? These percentages must sum to 100%.

In Experiment 4, a consensus was reached in 28 groups, and 105 (89.0%) of the 118 individuals’ final positions are located in the groups’ decision spaces. The correspondence of observed and predicted final opinions is *ρ* = 0.790, p < 0.0001, *n* = 354, and the correspondence of observed and predicted opinion changes is *ρ* = 0.806, p < 0.0001, *n* = 354. In Experiment 5, a consensus was reached in 31 groups, and 101 (85.6%) of the 118 individuals’ final positions are located in their groups’ decision spaces. The correspondence of observed and predicted final positions is *ρ* = 0.953, p < 0.0001, *n* = 472, and the correspondence of observed and predicted opinion changes is *ρ* = 0.814, p < 0.0001, *n* = 472.

## Discussion

We summarize our conclusions, and elaborate their implications, as follows. There are natural mathematical structures in group decision-making on resource distributions. (i) Geometric decision spaces are automatically generated in groups with heterogeneous initial positions on how a finite number of units of a resource should be distributed among options. The automatic construction of these decision spaces is a realization of a bounded rationality that sets up a circumscribed subspace of feasible resource distributions within the space of all possible distributions. A group with low variance initial allocation preferences will generate a smaller subspace of feasible distributions than a group with heterogeneous initial preferences. The decision space associated with a group’s array of initial positions can only include all possible distributions if it includes a set of individuals with an exhaustive set of possible extremal initial allocation positions. We note that the framework of formal optimization also has the feature of bounded rationality when it includes agreed upon min-max constraints on the amounts of a resource that may be allocated to each option. (ii) The quantitative values of individuals’ initial opinions become visible to other group members only if they are displayed by the individuals who hold them. The geometry of the implicit decision space polytope is quite complicated, and the individuals can hardly conceive it without special software. Yet, we find that in the absence of given or demanded explicit constraints, as in experiments 4–5, individuals’ settled final distribution opinions are usually located in the implicit group-specific subspace generated by their members’ initial min-max opinions on the amounts of a resource that should be allocated to each option. Remarkably, in these data, this tendency is not substantially lower than that observed in experiments 2–3, where there were given or demanded constraints. (iii) Our findings on the predictions of the Friedkin-Johnsen model suggest that groups’ final distribution opinions are usually constrained to their group-specific decision spaces because individuals update their opinions on the basis of a weighted averaging of their own and others’ displayed opinions. If all initial opinions are displayed, then weighted averaging, whether in the form specified by Friedkin-Johnsen or other forms, will not generate positions that are outside the convex hull of a group’s initial opinion array. Weighted averaging is consistent with opinion changes to extremal positions that are on vertices of the convex hull of initial opinions. The development of predictive generalized models, which allow opinion changes to positions outside the convex hull of initial opinions, is a topic of interest in the network science field on opinion dynamics. It is an open question whether such out-of-bounds events can be predicted.

The broad implication of the above is that, in these data, decision-making groups do not appear to be systems of bewildering confusion or disorder. A network science on small group decision-making appears surprisingly feasible. There are two main limitations of our results and interpretations. (i) Our experiments have not dealt with decision-making groups in field settings, where influence systems are subject to exogenous disturbances. (ii) Our measure of the influence network of a group is obtained from individuals’ post discussion reports of the extent to which their opinions were influenced by the opinions of other group members and the relative weights of each group member on their opinions. Addressing this limitation requires the development of technology that can process conversations and extract the relative weights that are the components of a weighted averaging update mechanism. Addressing these limitations toward a robust mathematical model of groups’ resource distribution decisions would allow model-based analysis of the implications of properties of groups’ initial opinion arrays, the distributed knowledge among group members, and the topologies of groups’ influence networks. A key motivation for such research is that models of optimal decisions begin with the definition of set of quantifiable givens (variables, constraints, and objectives) without specifying how these givens are obtained. Often, depending on the complexity of a problem, a collaborative group is involved in determining their definitions as prelude to an algorithmically obtained solution. Whether machine learning algorithmic definitions of optimization problems should replace human group definitions of optimization problems is an open question, particularly in the case of complex multi-objective problems that are attentive to the sort of qualitative concerns (distributive justice values and social costs) mentioned in our introduction. Elaborating the antecedents of the givens of optimization problems, and advancing knowledge on when formal optimization should or should not be relied upon, thus appear as central concerns.

## Materials and Methods

The University of California, Santa Barbara Institutional Review Board approved the study, and all subjects provided written informed consent. The subjects were recruited from the undergraduate subject pool of the Department of Psychological & Brain Sciences. All research was performed in accordance with relevant guidelines/regulations.

### Design and Measures

The data on the five experiments were obtained from three *disjoint* samples of subjects. Each group of 3–4 subjects were seated at a large table. No experimental manipulations or deceptions were involved. A problem was posed to the group. (1) Subjects privately recorded their initial positions on the problem. (2) A group discussion was then opened with the instruction: “Discuss the problem with the other members of your group. The goal is to reach an agreement. However, the conversation that you will have may or may not lead you to alter your initial answer, and you may not come to an agreement as a group.” (3) Upon concluding the discussion, subjects privately recorded their final position on the problem and their distribution of weights to other group members. On the latter, they were instructed as follows: “Imagine that you have been given a total of 100 chips. Distribute these chips to indicate the relative importance of each member to determining your own final answer on this problem. The number of chips that you allocate to a particular member should indicate the extent to which that member provided information that you personally found useful and caused you to modify your approach to the problem. The number of chips that you allocate to yourself should indicate the extent to which your final answer was not affected by the conversation. If the conversation had no influence on you, then put 100 beside your own sign (name). If the conversation caused you to abandon your approach to the problem, then put 0 beside your own sign, and allocate all the chips to one or more of the other members. If you did not entirely abandon your own approach to the problem, then put a number greater than 0 beside your sign and allocate the remainder to one or more others.” Subjective self-reported weights are as close as we currently can get to the construct definition of the cognitive algebra involved in individuals’ opinion revisions. (4) The experiment was then concluded or another issue was posed to the same group. A group had up to 30 minutes to respond to each issue. Thus, the design provides (i) a measure of individuals’ independent initial opinions, that is the *n* × *m* matrix **X**(0), (ii) a measure of their settled final opinions, that is the *n* × *m* matrix **X**(∞), and (ii) a measure of the weights in the convex combination mechanism by which each individual is integrating information on other individuals’ displayed opinions, that is, the matrix **W**.

### Statistical Analysis

The evaluative issue is whether individuals’ observed changes of opinion and settled opinions are consistent with the assumption of a weighted averaging mechanism of opinion updating. Observed opinion changes have signs and magnitudes. Evidence of a weak linear correspondence of predicted and observed opinion changes would erode the hypothesis of any natural meshing of a group decision space and a weighted averaging mechanism of opinion updating that operates to constrain revised opinions to positions located in the decision space. Such a mechanism would explain why final opinions are usually located in the convex hulls of groups’ initial opinion arrays. Given measures of a group’s **X**(0) and **W**, there are no model-intrinsic unknown parameters that require statistical estimation. Hence, no parameter estimation (optimizing the fit of predictions) is involved. Predicted opinion changes and settled opinions are obtained deterministically from the Eq. 5 dynamical system. Prediction errors have two sources (construct measure error or misspecification of the influence mechanism) in groups that are not subject to exogenous disturbances during the influence process. The statistical analysis is oriented to the question of whether the observed final opinions and opinion changes are consistent with the assumption of a convex combination mechanism that constrains revised opinion to the convex hull of a group’s initial opinion array. We use the direction and magnitude of the correlation coefficient *ρ*, and its statistical significance, to evaluate this question.

## Supplementary information

Mathematical Structures in Group Decision-Making on Resource Allocation Distributions. Supplementary Information.

## Data Availability

All data used in this analysis are available from the corresponding author on reasonable request.
