# Supplementary material for: Mathematical Structures in Group Decision-Making on Resource Allocation Distributions
Source: Sci Rep. 2019 Feb 4;9:1377. doi: 10.1038/s41598-018-37847-2 (PMC6361985; doi:10.1038/s41598-018-37847-2)
Supplement: Supplementary file 1 — Mathematical Structures in Group Decision-Making on Resource Allocation Distributions. Supplementary Information. [file 41598_2018_37847_MOESM1_ESM.pdf]

Mathematical Structures in Group Decision-Making on Resource Allocation Distributions. Supplementary Information.

Noah E. Friedkin[*,+], Anton V. Proskurnikov[+], W. Mei[+], and Francesco Bullo[+]

[*]Corresponding author.
[+]E-mails: friedkin@soc.ucsb.edu, anton.p.1982@ieee.org, meiwenjunbd@gmail.com, bullo@engineering.ucsb.edu

ABSTRACT

This supplement includes the properties of the Friedkin-Johnsen opinion dynamics model, and its generalization to multiple or multidimensional issues. We also provide details of statistical analysis, reported in the main paper.

The model definition

The Friedkin-Johnsen (FJ) opinion dynamics model is a generalization of the seminal French-Harary-DeGroot (30-32) model. Its motivation is the empirical observation that groups often do not reach consensus as predicted by the French-Harary-DeGroot influence mechanism in spite of strongly connected aperiodic interaction graphs. The generalization introduces ongoing levels of stubborn attachments to initial opinions. In this generalization, individuals' self-weights $0 \le w_{ii} \le 1$ correspond to their levels of anchorage on initial positions and the extent to which they are open or closed to the interpersonal influence of other individuals. As in the French-Harary-DeGroot model, the influence process is specified by discrete-time iterations of weighted averaging ("pooling") of the opinions

$$\begin{gathered} x_i(k+1) = a_{ii}\sum_{j=1}^{n} w_{ij}x_j(k) + (1-a_{ii})x_i(0), \quad i=1,\ldots,n, \;\; k=0,1,\ldots, \\ a_{ii} = 1-w_{ii},\; 0 \le w_{ij} \le 1,\; \sum_{j=1}^{n} w_{ij} = 1 \quad \forall i,j. \end{gathered} \tag{S1}$$

where $x_i(0) \in \mathbb{R}$ is the initial position of the group member i on an issue. The self-weights $\{w_{11},\ldots,w_{nn}\}$ may be heterogeneous. Individual i with $w_{ii} = 1-a_{ii} = 1$ is not subject to interpersonal influence: his/her position $x_i(k)$ is fixed at its initial value since $a_{ii}\sum_{j=1}^{n} w_{ij}x_j(k) = 0$. Individual i with $w_{ii} = 1-a_{ii} = 0$ and $(1-a_{ii})x_i(0) = 0$ at each time k updates his/her position to a weighted average of the time k positions of those group members to whom i has allocated influence,

$$x_i(k+1) = \sum_{j\ne i}^{n} w_{ij}x_j(k). \tag{S2}$$

For an individual i with $0 < w_{ii} < 1$, the initial position of the individual makes a continuing direct contribution $(1-a_{ii})x_i(0) = w_{ii}x_i(0)$ to any iteration of the influenced position of i. This contribution depends on extent to which the individual is open or closed to interpersonal influence. In this case, the $a_{ii}w_{ii} = (1-w_{ii})w_{ii} > 0$ value is the weight that i allocates to his or her own updated positions during the interpersonal influence process. This weight varies from 0 to 0.25 (achieved for $a_{ii} = w_{ii} = 0.5$) and corresponds to i's resistance to opinion change per se. Thus, the system of equations for the influence system on a specific issue is described by the matrix equation

$$\mathbf{x}(k+1) = \mathbf{AWx}(k) + (\mathbf{I}-\mathbf{A})\mathbf{x}(0), \;\; k=0,1,\ldots, \tag{S3}$$

where $\mathbf{A} = \operatorname{diag}[a_{11},a_{22},\ldots,a_{nn}]$ is a diagonal matrix, with $a_{ii} = 1-w_{ii}$ values on the main diagonal and zeros elsewhere, and $\mathbf{I}$ is the identity matrix (with ones on the main diagonal and zeros elsewhere). Note that $\mathbf{A}$ is determined by group members' self-weights in $\mathbf{W}$, and that the matrix $\mathbf{W}$ is a group-level construct derived from the mechanism specified by the theory. The mechanism is assumed to be the cognitive algebra of any individual's automatic information integration activity, and the

influence network is the social cognition structure assembled by the weights that individuals allocate to themselves and others in their information integration activity.

The process of interpersonal influence, which unfolds in the influence network $(\mathbf{A},\mathbf{W})$, may involve direct and indirect influences. The direct influences, at each time k, are described by (S1). Indirect interpersonal influences on an individual arise from the repetitive responses of individuals to the changing opinions of those to whom they have allocated direct influence. For instance, if a particular group member j, to whom i has allocated influence, has been affected by some other group member l, then l influences i indirectly. The state transition matrix $\mathbf{V}(k)=[v_{ij}(k)]$ of the discrete-time linear system (S3) defines the relative net influence of each group member j's initial opinion on the opinion of i at time $k+1$,

$$x_i(k+1)=a_{ii}\sum_{j=1}^{n}w_{ij}x_j(k)+(1-a_{ii})x_i(0)=\sum_{j=1}^{n}v_{ij}(k)x_j(0). \tag{S4}$$

The matrix $\mathbf{V}(k)$ may be obtained either with the matrix recursion, $\mathbf{V}(0)=\mathbf{I}$,

$$\mathbf{V}(k+1)=\mathbf{AWV}(k)+(\mathbf{I}-\mathbf{A}),\ \ \forall k\geq 0, \tag{S5}$$

or equivalently with the evolving matrix polynomial of walks in the graph of $\mathbf{AW}$,

$$\mathbf{V}(k)=(\mathbf{AW})^k+\left[\sum_{i=0}^{k-1}(\mathbf{AW})^i\right](\mathbf{I}-\mathbf{A}),\ \forall k\geq 1. \tag{S6}$$

Via induction on $k=0,1,\ldots$, it can be shown that the matrices $\mathbf{V}(k)$ are row-stochastic, that is $0\leq v_{ij}(k)\leq 1$ for all i, j and k and $\sum_{j=1}^{n}v_{ij}(k)=1$ for all i and k. Thus, on one-dimensional issues, all revised opinions are constrained to values in the interval defined by the two extremal min-max initial opinions of the group's members.

Basic Properties

The opinions $x_i(k)$ converge to steady values for any choice of $x_1(0),\ldots,x_n(0)$ if and only if the sequence $\{\mathbf{V}(k)\}_{k=0}^{\infty}$ converges, in which case the limit $\mathbf{V}=\lim_{k\to\infty}\mathbf{V}(k)$, referred to as the *control* matrix, describes the total (direct and indirect) influences of group member j's initial opinion on group member i's settled opinions on an issue

$$x_i(\infty)=a_{ii}\sum_{j=1}^{n}w_{ij}x_j(\infty)+(1-a_{ii})x_i(0)=\sum_{j=1}^{n}v_{ij}x_j(0)\quad\forall i$$

which can be rewritten in the matrix form as

$$\mathbf{x}(\infty)=\mathbf{AWx}(\infty)+(\mathbf{I}-\mathbf{A})\mathbf{x}(0)=\mathbf{Vx}(0). \tag{S7}$$

One may consider v_{ij} as the equilibrium relative total contribution of group member j's initial opinion to the settled opinion of group member i. Thus, each group member has a mean relative influence centrality, that is,

$$\bar{c}_i=\frac{1}{n}\sum_{j=1}^{n}v_{ji}, \tag{S8}$$

where each v_{ji} is the total relative influence of i's initial opinion on j's equilibrium opinion. Since $\mathbf{V}$ is a stochastic matrix, the mean influence centralities $\bar{c}_i$ sum to 1. In the special case of an $\mathbf{A}=\mathbf{I}$, the influence centrality in (S8) is equivalent to eigenvector centrality. In the special case of an $\mathbf{A}=\alpha\mathbf{I}$, $0<\alpha<1$, it is equivalent to PageRank centrality (20).

Convergence criteria

Necessary and sufficient conditions for the convergence of $\mathbf{V}(k)$ are covered in (20,29). It appears that $\mathbf{V}(k)$ converges if and only if $\lim_{k\to\infty}(\mathbf{AW})^k$ exists (such matrices are sometimes called *regular*). Violations of this regularity property are exceptional; it fails only in presence of some "closed" community in the network, whose members' opinions obey the French-Harary-DeGroot model with a periodic influence graph and are completely independent on the positions of the remaining individuals (18). Two *sufficient* conditions for the regularity of $\mathbf{AW}$ apply to the most interesting case where $\mathbf{A}\neq\mathbf{I}$ (that is, when the FJ model does not reduce to the French-Harary-DeGroot model). One sufficient condition is *strong connectivity* of the influence network, that is, the existence of mutual influence (direct or indirect) between opinions of any two individuals. The other sufficient condition

is $0 < \mathbf{A} < \mathbf{I}$ in which case individuals' levels of closure-openness are $0 < a_{ii} = 1 - w_{ii} < 1$ for all i. In both situations the matrix $\mathbf{AW}$ proves to be *Schur stable* (all eigenvalues are less than 1 in modulus and $\lim_{k\to\infty}(\mathbf{AW})^k = \mathbf{0}$), entailing that

$$\mathbf{V} = \left[\sum_{k=0}^{\infty}\left(\mathbf{AW}\right)^k\right]\left(\mathbf{I}-\mathbf{A}\right) = (\mathbf{I}-\mathbf{AW})^{-1}(\mathbf{I}-\mathbf{A}). \tag{S9}$$

A useful practical and remarkable property of the model is that (S9) is well-behaved when $\lim_{k\to\infty}(\mathbf{AW})^k \neq \mathbf{0}$ and $(\mathbf{I}-\mathbf{AW})^{-1}$ may not exist. It appears (18) that

$$\mathbf{V}_\alpha = \left[\sum_{k=0}^{\infty}\left(\alpha\mathbf{AW}\right)^k\right]\left(\mathbf{I}-\alpha\mathbf{A}\right), \quad 0 < \alpha < 1, \tag{S10}$$

is not ill-conditioned as $\alpha \approx 1$, converging to the control matrix

$$\mathbf{V} = \lim_{\alpha\to 1}\mathbf{V}_\alpha = \lim_{\alpha\to 1}\left(\mathbf{I}-\alpha\mathbf{AW}\right)^{-1}\left(\mathbf{I}-\alpha\mathbf{A}\right). \tag{S11}$$

Mathematical analysis shows that FJ predictions are robust under relaxations of the simplifying assumptions of synchronous opinion updates (29,36,37) and time-invariant $\mathbf{W}$ (38).

Tests

Experiments on groups of human subjects have evaluated the model's predictive accuracy on quantitative issues of judgment for which there are no true or false numerical positions (27,33,39-41). The designs of these experiments include groups in face-to-face interaction (18,39) and groups in which interaction is constrained by different telephonic communication structures that prohibit direct conversations among particular pairs of individuals and allow random dyadic conversations of varying length (27,33,39,40). Most recently, it has been applied and evaluated on quantitative intellective issues for there are true or false numerical positions (18).

Multiple or multidimensional issues

The influence system on a single or one-dimensional issue is easily generalized to a system of influences on multiple or multidimensional issues. The vector $\mathbf{x}(0)_{n\times 1}$ is replaced with a $\mathbf{X}(0)_{n\times m}$ matrix in which the m columns are different issues or dimensions of one issue. Starting at $\mathbf{X}(0)_{n\times m}$, the matrix of multidimensional opinions $\mathbf{X}(k)_{n\times m}$ evolves as

$$\mathbf{X}(k+1) = \mathbf{AWX}(k) + (\mathbf{I}-\mathbf{A})\mathbf{X}(0). \tag{S12}$$

This generalization extends the domain of applications of the model. For example, $\mathbf{X}(0)_{n\times 3}$ may be individuals' simultaneous initial attitudinal orientations to three objects, or to three dimensions of one object. An interesting application of this generalization is to a $\mathbf{X}(0)_{n\times m}$ matrix in which m is a number of discrete alternative choices or options that are being appraised by a group. The appraisal takes the form of a relative weighting of the alternatives, i.e, when $x_{i1}, x_{i2}, \ldots, x_{im}$ are nonzero values that sum to 1 for each i. In this case, $\mathbf{X}(0)$ is a single multidimensional issue that is defined as the relative weighting of m alternatives. The allocated weights to the m alternates not only display the qualitative preference ordering of the alternatives for each individual, but also the quantitative strengths of these preferences. For example, on three alternatives, two individuals' preferences might be $\{x_{i1} = 0.40, x_{i2} = 0.35, x_{i3} = 0.25\}$ and $\{x_{j1} = 0.80, x_{j2} = 0.15, x_{j3} = 0.05\}$. Their preference *orderings* are identical, but they clearly importantly differ in their appraisals of the alternatives. Modifications of individuals' positions on such an issue preserve the property of the appraisal structure; that is, each row of $\mathbf{X}(k)$, $k = 1, 2, \ldots$ is composed of nonzero values that sum to 1. In this article, we apply the model to a $\mathbf{X}(0)_{n\times m}$ matrix in which m is a number of discrete alternative options over which a quantity is distributed. On multiple or multidimensional issues, all revised opinions are constrained to the convex hull associated with the group's $\mathbf{X}(0)$ array of initial opinions.

The model in (S12) assumes that m dimensions of the opinion evolve independently, that is, $\mathbf{X}(k)$ could be assembled by a sequentially processing each of its columns as a single issue. This entails the following properties:

1. all rows $\mathbf{X}(k)$ are in the m-dimensional convex hull spanned by the rows of $\mathbf{X}(0)$;
2. if the entries $x_{ij}(0)$, $j = 1, \ldots, m$, in each row of $\mathbf{X}(0)$ satisfy $x_{ij}(0) \geq 0, \sum_{ij}^{m} x_{ij}(0) = b > 0$, the same conditions hold for each row of $\mathbf{X}(k)$;
3. if rows of $\mathbf{X}(0)$ are ordered $x_{i1}(0) \leq x_{i2}(0) \leq \cdots \leq x_{im}(0)$, then the same ordering holds for the rows of $\mathbf{X}(k)$.

To prove these properties, we need some basic facts from convex geometry. By definition, a *convex combination* of several points $\mathbf{p}_1,\dots,\mathbf{p}_s \in \mathbb{R}^m$ is a weighted average

$$\begin{aligned} &\mathbf{p} = w_1\mathbf{p}_1 + \alpha_2\mathbf{p}_1 + \dots + w_s\mathbf{p}_s \\ &\text{where} \quad w_i \geq 0 \forall i, \ \sum_i w_i = 1. \end{aligned} \tag{S13}$$

The set of *all* convex combinations (S13) (corresponding to all possible choices of the weights w_i) is called the *convex hull* spanned by $\mathbf{p}_i$ and is denoted by conv $\{\mathbf{p}_i\}_{i=1}^s$ or conv $\{\mathbf{p}_1,\dots,\mathbf{p}_s\}$.

A set $M \subseteq \mathbb{R}^m$ is said to be *convex* if with any two points $\mathbf{p}_1,\mathbf{p}_2 \in M$ it contains their convex hull, that is, the line segment connecting $\mathbf{p}_1$ and $\mathbf{p}_2$ lies entirely within M

$$\alpha\mathbf{p}_1 + (1-\alpha)\mathbf{p}_2 \in M, \ \forall \mathbf{p}_1,\mathbf{p}_2 \in M, \ \forall \alpha \in [0,1].$$

Using induction on $s \geq 2$, it can be shown that the same condition automatically holds for any set of s vectors: if M is convex and $\mathbf{p}_1,\dots,\mathbf{p}_s \in M$, then $C = \text{conv}\ \{\mathbf{p}_i\}_{i=1}^s \subseteq M$. It can be shown that the convex hull C is convex, being thus the *minimal* convex set that contains the points $\mathbf{p}_1,\dots,\mathbf{p}_s$. Other examples of convex sets are:

1. simplex: $M = \{(x_1,\dots,x_m) : x_i \geq 0, \sum_i x_i = b > 0\}$;
2. closed ball: $M = \{(x_1,\dots,x_m) : \sum_i x_i^2 \leq R^2\}$;
3. polyhedron (in general, unbounded): $M = \{(x_1,\dots,x_m) : xA \leq b$, where A is a matrix and b is a row vector;
4. set of preordered vectors: $M = \{(x_1,\dots,x_m) : x_1 \leq x_2 \leq \dots \leq x_m\}$.

For the multidimensional FJ model in (S12), the following lemma holds.

Lemma. If each row $x_i(0) = (x_{i1}(0),\dots,x_{im}(0))$ of the matrix $\mathbf{X}(0)$ belongs to a *convex* set $M \subseteq \mathbb{R}^m$, the same holds for $\mathbf{X}(k)$ for all $k \geq 0$.

Proof. The statement is proved via induction on k. The induction base $k = 0$ is obvious. Assuming that the statement has been proved $k \leq k_0$, we now prove it for $k = k_0 + 1$. Denoting the ith row of $\mathbf{X}$ by $\mathbf{x_i}$, (S12) entails that

$$\mathbf{x}_i(k_0+1) = a_{ii}\sum_{j=1}^{n} w_{ij}\mathbf{x}_j(k_0) + (1-a_{ii})\mathbf{x}_i(0), \tag{S14}$$

that is, each row of $\mathbf{X}(k+1)$ is a convex combination of the rows of $\mathbf{X}(k)$ and one row of $\mathbf{X}(0)$, which row vectors belong to M due to the induction hypothesis. Since M is a convex set, $\mathbf{x}_i(k_0+1)$ also belongs to M for all $i = 1,\dots,n$ thanks to (S13). This proves the inductive step.■

The aforementioned properties 1-3 of the FJ model are nothing else than special cases of Lemma, where M stands respectively for the convex hull, spanned by the rows of $\mathbf{X}(0)$ (Property 1), the simplex $\{(x_1,\dots,x_m) : x_i \geq 0, \sum_i x_i = b > 0\}$ (Property 2) and the set of ordered vectors $\{(x_1,\dots,x_m) : x_1 \leq x_2 \leq \dots \leq x_m\}$ (Property 3).

Statistical Analysis

Given measures of the FJ $\mathbf{X}(0)$ and $\mathbf{W}$ construct, note that there are no model-intrinsic unknown parameters that require statistical estimation. The theory gives us the necessary and sufficient constructs, and with measures of these constructs we generate a prediction. Under the assumptions of the FJ influence system model specification, if these two measures are error free, then there should be an exact correspondence of individuals' observed and predicted final opinions, $\mathbf{X}(\infty) = \mathbf{V}\mathbf{X}(0)$, because $\mathbf{V}$ is strictly determined by $\mathbf{W}$. Recall that $\mathbf{A}$, which enters into the derivation of $\mathbf{V}$, is also strictly determined by $\mathbf{W}$. Similarly, there should be an exact correspondence of observed and predicted opinion changes, $\mathbf{X}(\infty) - \mathbf{X}(0)$ and $\hat{\mathbf{X}}(\infty) - \mathbf{X}(0)$, respectively. This correspondence is, however, inevitably attenuated by errors in measurements and model specifications. The advancement of a network science modeling of opinion dynamics is currently limited by the absence of a technology that provides a more direct measurement of the causal basis of these dynamics and, in particular, a more direct measurement of the weights in the convex combination mechanism that is the widely assumed "cognitive algebra" of iterated opinion update processes. The statistical analysis is oriented to the question of whether the observed final opinions and opinion changes are consistent with the assumption of a convex combination mechanism that constrains revised opinion to the convex hull of a group's initial opinion array. Thus, we have reported findings on (i) the extent to which individuals' final opinions are located in their group's convex hull and (ii) the strength of linear correspondence of observed and predicted final opinions and opinions changes. The findings on the correspondence are based on reshaping of the $n \times m$ arrays of observed and predicted final opinions and opinion changes into $mn \times 1$ vectors. Below we display the resulting scatter plots on the associations reported in Experiments 2-5, and Experiment 3's Table 1.

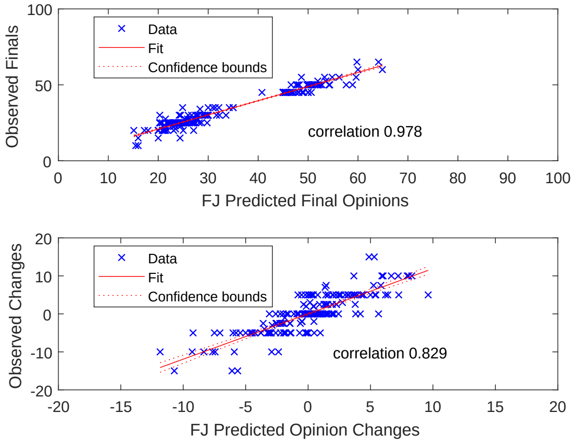

Figure S1. Experiment 2. Opinions on preferred percentages constrained by IOM Acceptable Macronutrient Distribution Range (AMDR): 45-65% Carbohydrates, 10-35% Protein, and 20-35% Fat. The association of observed and FJ predicted final opinions, and association of observed and FJ predicted opinion changes.

Ideal Positions	Min-Max Constraints		
	Disagreement	Consensus	Totals
Disagreement	8	2	10
Consensus	6	7	13
Totals	14	9	23

Table 1. Fisher's exact test $P > 0.05$.

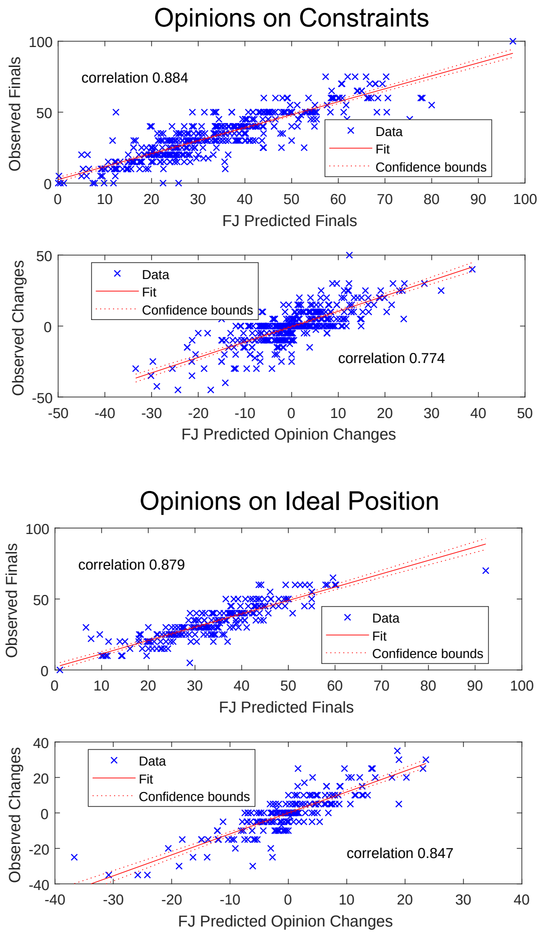

Figure S2. Experiment 3. Opinions on constraints and ideal percentages of total food consumption that should be based on Fruits/Vegetables, Grains, and Meats. The association of observed and FJ predicted final opinions, and association of observed and FJ predicted opinion changes.

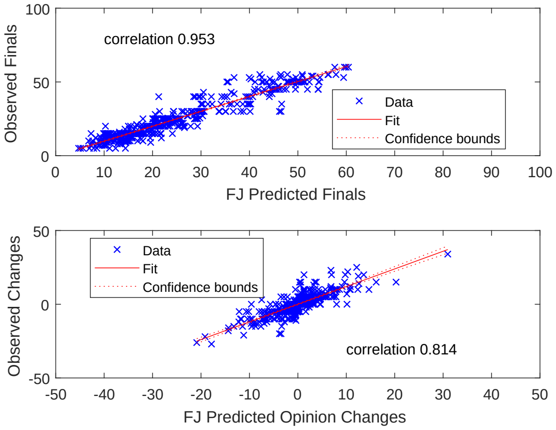

Figure S3. Experiment 4 on a state tax revenue allocation issue. The association of observed and FJ predicted final opinions, and the association of observed and FJ predicted opinion changes.

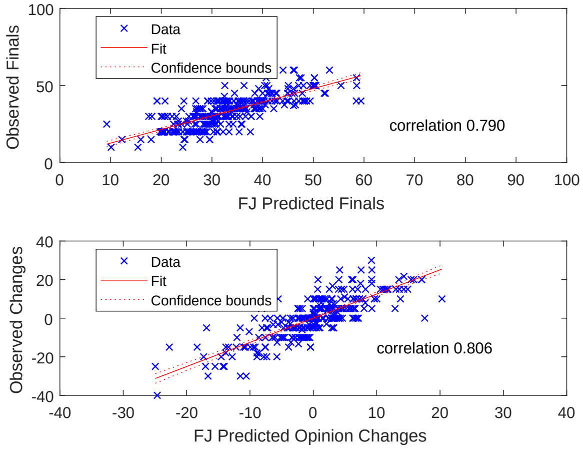

Figure S4. Experiment 5 on the allocation of permissions to recruit new faculty on a university campus. The association of observed and FJ predicted final opinions, and the association of observed and FJ predicted opinion changes.
